# Awareness and attitudes towards ayurveda in chronic disease management and related knowledge gaps among UK healthcare professionals: an analytical cross-sectional survey

**DOI:** 10.1186/s12906-026-05283-9

**Published:** 2026-03-06

**Authors:** Vyshna Ravindran, Rajeev Gupta, Sunil Bhandari, Felicity Evison, Sheila Greenfield, Kanta Kumar

**Affiliations:** 1 College of Medicine and Health, School of Health Sciences, University of Birmingham, Birmingham, UK; 2 Vaidyaratnam P.S Varier Ayurveda College, Kottakkal, India; 3 University of Sheffield, Sheffield, UK; 4 Barnsley Hospital NHS Foundation Trust, Sheffield, UK; 5 Hull University Teaching Hospitals NHS Trust, Hull, UK; 6 Institute of Translational Medicine, University Hospitals Birmingham NHS Foundation Trust, Birmingham, UK; 7 Department of Rheumatology, Royal Wolverhampton NHS Foundation Trust, Wolverhampton, UK

## Abstract

**Background:**

Chronic diseases account for 74% of global deaths. In the UK, multimorbidity prevalence is projected to nearly double by 2035. Addressing this burden requires a holistic, multidisciplinary approach that considers genetic, lifestyle, environmental, and psychosocial factors. The increasing focus on holistic care has revived interest in traditional and complementary healthcare practices, with a 2013 survey indicating that 23.6% of the UK’s ethnic population utilizes these modalities. Among these practices, Ayurveda has gained significant global traction. Despite the growing popularity of Ayurveda, discussions between patients and healthcare professionals (HCPs) remain limited. Consequently, patients often explore Ayurveda centres independently, risking unreliable sources that could have serious consequences on their health. Opening conversations about Ayurveda is crucial to ensure patients are well-informed. These conversations largely depend on the awareness, attitudes, and having the confidence and the ability to signpost patients to reputable Ayurvedic centres.

**Methods:**

The study utilized a cross-sectional survey to assess UK HCPs’ awareness, attitudes, and training needs on Ayurveda. After a pilot test, an anonymous Google Form was distributed, yielding 147 responses. Cronbach’s alpha for the questionnaire was 0.9261, indicating high internal consistency. Descriptive statistics, Chi-Square Test, and Fisher’s Exact Test were used for data analysis.

**Results:**

Of 147 surveyed HCPs, 10.2% were very familiar with Ayurveda, 46.3% somewhat familiar, and 43.5% had little to no familiarity. Herbal medications (59.6%) and dietary interventions (41.1%) were most recognized. Only 10.9% fully understood Ayurveda’s known safety and effectiveness. Most (80.3%) felt unprepared to advise patients on Ayurveda, and majority (83%) had no formal training.

Regarding attitudes, 46.2% supported including Ayurveda as a treatment option, and 82.3% thought it should be regulated by the Medicines and Health Products Regulatory Agency (MHRA) or the NHS. The majority (70.7%) favoured collaboration with Ayurvedic practitioners. Attitudes about Ayurveda’s role in chronic disease varied, with 47.7% believing it could help those not benefited by conventional medicine. A strong majority (83.7%) supported patient education on its benefits and risks, and most (86.4%) willing to learn more.

In terms of patient engagement, nearly half (47.6%) refrained from offering definitive advice on Ayurveda, and only a few (8.8%) referred patients to Ayurvedic practitioners. However, most (84.4%) supported interprofessional cooperation. Key challenges to integration included lack of scientific evidence (55.1%) and limited understanding (44.2%), with many advocating for research (51.7%) and education (45.6%).

Ethnicity significantly influenced responses (p<0.001), with Asians showing familiarity but limited understanding, Black respondents displaying more knowledge and support for integration, and White respondents expressing scepticism and a need for regulation.

**Conclusion:**

The survey highlights significant gaps in awareness and understanding of Ayurveda among UK healthcare professionals, alongside varying attitudes toward its integration in chronic disease management. Most HCPs expressed support for collaboration with Ayurvedic practitioners and for educating patients about its benefits and risks. These findings underscore the need for enhanced education and structured training programs for HCPs to enable safe and informed discussions about Ayurveda in clinical practice. In response to the identified gaps, an educational video was subsequently developed by the University of Birmingham for HCPs to address knowledge gaps and support informed patient decisions on Ayurveda.

**Supplementary Information:**

The online version contains supplementary material available at 10.1186/s12906-026-05283-9.

## Introduction

 The global burden of chronic conditions is rising, accounting for 74% of all deaths worldwide [1]. In Europe, they are the leading cause of mortality, responsible for 77% of disease burden and 86% of deaths [2]. Chronic conditions significantly reduce quality of life [3] and impose substantial economic costs [1]. In the United Kingdom (UK), multimorbidity is increasing, with those experiencing four or more disorders expected to nearly double by 2035 [4]. Preventing and managing chronic conditions is particularly challenging due to their multifactorial nature. Therefore, addressing chronic conditions requires a holistic, multidisciplinary approach that considers genetic, lifestyle, environmental, and psychosocial factors [5]. 

The rise in interest in holistic care has led to the increase global use of complementary and alternative medicine (CAM), with the World Health Organisation reporting 70–80% usage in developed countries [6]. The global market for CAM was valued at USD 144.68 billion in 2023, expected to grow at 25.3% annually from 2024 to 2030 [7]. In the UK, a 2013 survey reported a one-year usage rate of CAM of 41.1% and a lifetime prevalence of 51.8% [8] The UK exhibits significant diversity, with individuals of Asian backgrounds, constituting 5.76 million, or 8.6%, of the overall population [9–11]. Ayurveda, a traditional system of medicine that originated in India, has gained traction within this population and more broadly across the UK [12] The increased interest can be attributed to factors such as holistic philosophies, the availability of information on the internet, and public exposure to CAM in diverse cultures [12]. 

Despite this rising global popularity, there remains a noticeable gap in opening the discussion in consultation on Ayurveda between patients and healthcare professionals (HCPs), especially in western countries. As a result, patients often explore Ayurvedic centres on their own, sometimes risking unreliable and unauthorised sources that could have serious consequences on their health. Studies in the United Arab Emirates (UAE) [13], South Africa [14], and Karachi [15] reveal that CAM users often rely on personal recommendations over professional advice, disregarding medical guidance. This underscores the need for HCPs to be well-informed about CAM including Ayurveda to ensure patient safety, prevent harmful treatments, and foster open dialogue for better patient-physician relationships. However, the success of these conversations depends heavily on HCPs’ awareness, attitudes, and training to signpost patients to reputable Ayurvedic centres.

In the UK, Ayurveda is classified under CAM and does not have statutory regulation or formal recognition within the NHS. Ayurvedic practitioners are not state-registered healthcare professionals, and their scope of practice is largely limited to lifestyle advice, dietary recommendations, and provision of herbal formulations. They cannot prescribe conventional medications or order investigations available to NHS doctors. Oversight is provided mainly through voluntary registration with professional bodies which set codes of conduct but lack statutory authority. Herbal products used in Ayurveda are subject to the UK Medicines and Healthcare products Regulatory Agency (MHRA) under the Traditional Herbal Registration (THR) scheme, which regulates safety and quality but not clinical efficacy. Consequently, Ayurveda remains available privately rather than as a commissioned NHS service, with limited integration into mainstream healthcare.

Previous UK studies have explored HCPs’ knowledge and attitudes toward CAM. A study among physicians in the UK showed generally positive attitudes among physicians, especially in palliative care, rehabilitation, nuclear medicine, and genito-urinary medicine [16]. A qualitative study of NHS academic doctors, the latest to have explored this area, revealed mixed views, with advocates supporting patient choice but calling for robust evidence, while sceptics cited cost and resource concerns [17]. Limited formal CAM training exists, though HCPs show interest in learning. A survey found 87% of CAM-using physicians lacked formal training, yet many supported CAM integration and its inclusion in medical education [16]. Further, a systematic review on the perception of CAM found that practitioners open to CAM expressed willingness to learn for patient benefit [18]. 

Two studies have specifically examined the knowledge and attitudes regarding Ayurveda outside the UK. An Indian study among 100 allopathic resident doctors found 48% knew Ayurvedic concepts, 81% were unfamiliar with Panchakarma (a specialized Ayurvedic therapeutic procedure that focuses on detoxification and rejuvenation by systematically eliminating accumulated toxins from the body), and 92% called for more scientific validation. While 60% felt integration could improve satisfaction, 80% opposed mandatory training [19]. In Bangladesh, most doctors treated patients with Ayurveda, supported its recognition as a specialty, and highlighted gender and age influences, with older and female doctors showing greater support for its efficacy and promotion [20]. 

However, the perspectives of HCPs in the UK remain largely unexplored. Given that UK HCPs serve a significant population of South Asian descent, among whom Ayurveda is widely used, understanding the extent to which UK HCPs engage in discussions about Ayurveda with their patients is critical. However, the absence of formal education on traditional systems such as Ayurveda within the UK medical curriculum makes these discussions challenging. This cross-sectional study therefore aimed to investigate UK HCPs’ awareness, attitudes, and training needs concerning the use of Ayurveda in managing chronic conditions.

## Methods

Study Design: Ethical approval for the study was obtained from the University of Birmingham Research Ethics Committee (Ethics number: ERN_1729-Oct2023). Our study employed STROBE guidelines for reporting the data. Existing validated tools like CAM Health Belief Questionnaire (CHBQ) [21] and Integrative Medicine Attitude Questionnaire (IMAQ) [22], focused on general CAM attitudes, were not suitable for the study’s specific aims. A custom questionnaire was hence developed through literature review, to assess UK HCPs’ awareness, attitudes, and training needs regarding Ayurveda. It was piloted with five HCPs to refine usability, layout, and clarity. The questionnaire was designed as a 15-minute anonymous Google Form.

Setting and Participants: The study targeted UK healthcare professionals, including doctors, nurses, and allied health practitioners, representing primary care, secondary hospitals, and private practices. Eligible participants were actively practicing HCPs in the UK. Informed consent was embedded in the Google Form, ensuring privacy and confidentiality. Participation was voluntary, with options to skip questions or withdraw at any time. Data collection spanned five months, from April to August 2024.

Variables: Potential confounders were educational level (formal healthcare education and CAM training) and work setting (type of healthcare organization and demographics). Effect modifiers included cultural background (self-reported ethnicity) and prior Ayurveda experience.

Bias: To enhance validity and reliability, potential biases were mitigated by piloting the questionnaire to address ambiguities, using standardised questions and validated scales, ensuring diverse sampling via professional organizations as well as local networks, and maintaining anonymity to reduce social desirability bias.

Sample size: We employed a non-probability convenience sampling strategy. Because distribution used multiple organisational mailing lists, newsletters, and forwarding/resharing by individual recipients, and because lists overlapped across organisations, a single definitive denominator (the total number of unique individuals who received the invitation) could not be established. Therefore, an exact response rate cannot be calculated. We received 147 valid responses during the study period.

Data sources/measurement: Data was collected via a literature-based questionnaire assessing UK HCPs’ awareness, attitudes, and training needs on Ayurveda for chronic conditions. Internal consistency, measured by Cronbach’s alpha, was 0.8758, 0.9232, and 0.8499 for awareness, attitudes, and training needs, respectively, with an overall value of 0.9261, indicating high reliability.

Statistical methods: Descriptive statistics summarised demographics and responses. Chi-Square tests were used to examine associations between categorical variables; when expected cell counts were < 5, Fisher’s Exact Tests were applied. The statistical test used for each p-value is indicated in Table 1 footnotes. Cronbach’s Alpha assessed questionnaire reliability. Statistical analyses were conducted in STATA (v15.1), with significance set at *p* < 0.01.

**Table 1:** Sociodemographic characteristics of participants

Variable	Attributes	Percentage
Age (years)	18–24	2%
	25–34	11.6%
	35–44	11.6%
	45–54	40.1%
	55–64	27.9%
	65–74	4.8%
	75 and older	2%
Gender	Male	34%
	Female	66%
	Non-binary	0%
	Prefer not to say	0%
Ethnicity	Caucasian	22.4%
	Black/Black British/African/Caribbean	16.3%
	Asian or Asian British	57.1%
	Mixed or Multiple ethnic groups	2%
	Other ethnic group	1.4%
	Prefer not to say	0.7%
Geographic location	North East	4.1%
	North West	23.8%
	Yorkshire and the Humber	40.1%
	East Midlands	2.7%
	West Midlands	12.2%
	East of England	1.4%
	London	1.4%
	South East	2.7%
	South West	4.8%
	Scotland	4.1%
	Wales	2.7%
	Northern Ireland	0%
Profession	Medical specialty doctor	55.8%
	General practitioner	26.5%
	Nurse	5.4%
	Pharmacist	6.1%
	Others	6.2%
Years of experience in healthcare	Less than 5 years	4.8%
	5–10 years	4.1%
	11–20 years	32%
	21–30 years	18.4%
	More than 30 years	40.8%
Type of healthcare setting	Secondary care	60.5%
	Primary care	30.6%
	Private practice	6.1%
	Other	2.8%

## Results

### Sociodemographic characteristics of participants

A total of 147 survey responses were received. Demographic analysis revealed a predominantly mid-to-late career group, with most participants aged 45–54 years (40.1%) and 55–64 years (27.9%). A significant proportion (40.8%) had over 30 years of healthcare experience and worked in secondary care settings (60.5%). The gender distribution included a significant proportion of females (66%) compared to males (34%). Ethnic diversity was notable and included Asian/Asian British (57.1%), Caucasian (22.4%), and Black/Black British/African/Caribbean (16.3%). Yorkshire and the Humber (40.1%) and the Northwest (23.8%) had the highest regional representation. Most participants (82.3%) had a medical background, comprising hospital-based specialists (55.8%) and general practitioners (26.5%). The specialists represented a wide range of fields, including Rheumatology, Paediatrics, Endocrinology, Gynaecology, Orthopaedics, Psychiatry, Anaesthesiology, Hepatology, Gastroenterology, Geriatrics, Nephrology, Neurology, Emergency Medicine, Cardiology, Haematology, and Ophthalmology. (Table 1).

### Awareness of UK HCPs on ayurveda for chronic condition management

Familiarity with Ayurveda: Among participants, 10.2% were very familiar with Ayurveda, 46.3% somewhat familiar, 32% not very familiar, and 11.6% not at all familiar. Recognised domains included herbal medications (59.6%) and dietary interventions (41.1%), with limited awareness of Panchakarma therapies (10.6%) and Ayurvedic surgical interventions (5%).

Perceived Safety and Effectiveness: Only 10.9% of participants felt they completely understood Ayurveda’s safety and effectiveness for chronic conditions. In contrast, 30.6% had minimal understanding, and 43.5% had no understanding. Diabetes (30.6%) and musculoskeletal conditions (27.2%) were identified as areas where Ayurveda might be most used and effective. Regarding research quality, only 5.4% rated it as high quality, while 28.6% rated it low, and 54.4% were unsure.

Access to Information and Training: Most participants (80.3%) felt inadequately prepared to counsel on Ayurveda. Access to reliable information was challenging for many, with 42.2% unsure how to access information on reputable centres and 37.4% found it difficult to have the information readily available. Sources of information on Ayurveda primarily came from medical journals (54.4%) and online resources (24.5%), with few using conferences (6.8%). Training in Ayurveda was rare, as 83% reported no formal education, and 61.9% were unfamiliar with legal and regulatory issues.

### Attitude of UK HCPs on ayurveda for chronic condition management

Towards Integration and Institutionalisation: Moderate support was observed for including Ayurveda in clinical discussions, with 46.2% favouring (12.2% strongly agree, 34% agree), though 45.6% remained neutral. On accessibility via NHS, 45.6% supported it (7.5% strongly agree, 38.1% agree), while 46.3% were neutral. Regulation by the MHRA (Medicines and Healthcare products Regulatory Agency) or NHS was supported by 82.3% (38.1% strongly agree, 44.2% agree), with 83.7% (37.4% strongly agree, 46.3% agree) concerned about risks from unregulated practice. Collaboration with Ayurvedic practitioners was supported by 70.7% (23.8% strongly agree, 46.9% agree), but personal willingness to engage was lower at 46.9% (13.6% strongly agree, 33.3% agree), with 45.6% neutral. Formal referrals between NHS and Ayurvedic practitioners had 45.6% support (12.9% strongly agree, 32.7% agree) and 42.9% neutrality.

In Chronic Condition Management: Support for ensuring access to qualified Ayurveda practitioners was high with 85% (38.1% strongly agree, 46.9% agree). Mixed beliefs were noted on Ayurveda’s role in chronic conditions: 41.5% agreed it could prevent them (19.7% strongly agree, 21.8% agree), 44.9% supported its role in management (11.6% strongly agree, 33.3% agree), and 38.1% agreed it addressed root causes (19.7% strongly agree, 18.4% agree). A significant number of participants (45.5%) believed that Ayurveda could improve the quality of life for patients (12.2% strongly agree and 33.3% agree), and 47.7% believed it could help those unresponsive to conventional medicine (11.6% strongly agree and 36.1% agree). Moderate support (44.2%) was noted for integrating Ayurveda into routine practice to enhance patient satisfaction (10.2% strongly agree, 34% agree), and 47.6% remaining neutral. A substantial 61.9% of participants believed Ayurveda can contribute to patient-centred care (13.6% strongly agree, 48.3% agree). The majority (85.7%) also agreed that more research is needed on the safety and efficacy of Ayurveda for managing chronic conditions (53.7% strongly agree, 32% agree). Similarly, 79% of participants supported allocating more resources to Ayurvedic research (46.3% strongly agree, 32.7% agree). Furthermore, 79.6% agreed that HCPs should collaborate with Ayurvedic practitioners to develop evidence-based guidelines (34% strongly agree, 45.6% agree), with 10.9% in disagreement. Key measures for safe and effective Ayurveda use in the UK included educating HCPs/patients (43.5%), research (40.1%), and developing guidelines/regulations (38.1%).

Education and training: There was strong support for informing patients about both the benefits and risks of Ayurveda, with 83.7% in agreement (44.9% strongly agree and 38.8% agree). Furthermore, 86.4% of participants were willing to learn more about Ayurveda (49% strongly agree and 37.4% agree), and 81.6% supported offering educational training on Ayurveda to HCPs (34% strongly agree and 47.6% agree).

### Training needs of UK HCPs on ayurveda for chronic condition management

HCPs’ Views on People’s Interest and Communication: People’s interest in Ayurveda was low, with only 7.5% of participants reporting frequent interest, while the majority perceived it as rarely (44.9%) or never (32.7%). When people expressed interest, 47.6% of HCPs refrained from offering advice, 23.8% provided information, and 7.5% referred to Ayurvedic practitioners. This hesitance may stem from a lack of confidence and knowledge, as the majority (55.1%) were uncomfortable discussing Ayurveda. Majority of HCPs (55.8%) never discussed Ayurveda’s benefits and an even higher percentage (61.2%) never mentioned its potential risks. Additionally, most HCPs (46.3%) never asked people about Ayurveda use. While 57.2% believed people disclosed its use occasionally or frequently, 42.8% thought it was rarely or never mentioned.

Referrals and Interprofessional Relations: Referrals to Ayurvedic practitioners were rare, with only 8.8% of HCPs having made such referrals. Among them, 46.9% cited non-response to conventional treatments as the reason of referral. The predominant barriers to referral included a lack of knowledge about Ayurveda (47.6%) and limited access to Ayurvedic practitioners (25.2%). Despite this, majority (84.4%) were open to collaboration with Ayurvedic practitioners. However, interprofessional communication about Ayurveda remained infrequent, with 80.3% of HCPs rarely or never discussing it with colleagues. Majority (62.6%) perceived occasional or frequent lack of acceptance among peers regarding Ayurveda in chronic condition management.

Perceived Challenges: The main challenge to integrating Ayurveda in UK chronic condition management was lack of scientific evidence (55.1%). Other challenges included limited understanding among HCPs (44.2%) and absence of a regulatory framework (25.2%). A significant majority (83%) found the current regulatory framework inadequate, raising concerns on patient safety. Moreover, 90.5% believed resources for integration were insufficient. To overcome these challenges, majority HCPs advocated for more scientific research (51.7%), regulatory development (45.6%), and education for HCPs (45.6%).

### Association between participant demography and responses

The data highlighted a strong association between ethnicity and UK HCP’s responses on awareness, attitudes and training needs related to Ayurveda.

#### Association between ethnicity and awareness of UK HCPs on ayurveda for chronic condition management

The survey identified a significant association (*p* < 0.001) between ethnicity and UK HCPs’ awareness of Ayurveda for chronic condition management. Asians reported the highest familiarity, with the majority (83.5%) either “Very familiar” or “Somewhat familiar”, compared to < 30% of Black participants and 10% of Caucasians. However, almost three-quarters of Asian participants (74.1%) reported minimal or no understanding of its clinical safety and efficacy, while, Black participants demonstrated the highest level of understanding, with 50% having either complete or partial knowledge. Caucasians expressed the highest level of scepticism about the quality of Ayurvedic research, with none rating it positively and over half considering it “Low quality”. They also displayed the lowest confidence in counselling on Ayurveda, with participants either disagreeing (51.5%) or strongly disagreeing (48.5%) about their preparedness.

Black participants showed the highest difficulty in finding information, with 45.8% finding it difficult and 16.7% finding it very difficult. Asian participants, despite cultural connections to Ayurveda, had the highest uncertainty about accessing information, with 55.3% unsure of how to find reliable sources, and 23.6% finding it either difficult or very difficult. Caucasian participants had no training in Ayurveda, while Black participants had the highest participation with 50% reporting some level of training. Caucasian participants were either not very familiar (51.5%) or not at all familiar (48.5%) with Ayurvedic legal issues, while majority (82.4%) of Asians also reported significant unfamiliarity despite cultural ties. (Table 2).

**Table 2:** Association between participant demography and responses

Association between ethnicity and awareness of UK HCPs on Ayurveda for chronic condition management					
Variable	Attributes (n)	Caucasian *N* = 33	Black *N* = 24	Asian *N* = 85	p-value
How familiar are you with Ayurveda?	Very familiar	0 (0.0%)	4 (16.7%)	11 (12.9%)	< 0.001
	Somewhat familiar	2 (6.1%)	3 (12.5%)	60 (70.6%)	
	Not very familiar	21 (63.6%)	13 (54.2%)	12 (14.1%)	
	Not at all familiar	10 (30.3%)	4 (16.7%)	2 (2.4%)	
which of the following domains of Ayurvedic intervention are you familiar with?	Herbal medications*	6 (18.2%)	2 (8.3%)	75 (88.2%)	< 0.001
	Medications with metals and minerals	2 (6.1%)	4 (16.7%)	10 (11.8%)	0.426
	Panchakarma therapies	0 (0.0%)	1 (4.2%)	12 (14.1%)	0.027
	Ayurvedic surgical interventions	0 (0.0%)	3 (12.5%)	4 (4.7%)	0.111
	Ayurvedic para-surgical interventions	1 (3.0%)	4 (16.7%)	5 (5.9%)	0.147
	Dietary interventions*	17 (51.5%)	10 (41.7%)	30 (35.3%)	0.268
	None of the above	8 (24.2%)	1 (4.2%)	5 (5.9%)	0.01
How much do you understand the efficacy and safety of Ayurveda in managing chronic conditions?	Completely understand	0 (0.0%)	6 (25.0%)	9 (10.6%)	< 0.001
	Partially understand	0 (0.0%)	6 (25.0%)	13 (15.3%)	
	Have minimal understanding	20 (60.6%)	9 (37.5%)	15 (17.6%)	
	Have no understanding	13 (39.4%)	3 (12.5%)	48 (56.5%)	
In which chronic conditions do you think Ayurveda may be effective in managing	Cardiovascular diseases (including heart disease and stroke)*	3 (9.1%)	2 (8.3%)	22 (25.9%)	0.039
	Chronic respiratory diseases (such as chronic obstructive pulmonary disease - COPD)*	2 (6.1%)	3 (12.5%)	18 (21.2%)	0.123
	Diabetes (Type 1 and Type 2)*	4 (12.1%)	1 (4.2%)	38 (44.7%)	< 0.001
	Musculoskeletal conditions (such as arthritis)*	7 (21.2%)	2 (8.3%)	30 (35.3%)	0.021
	Neurological diseases (such as Parkinson’s disease and multiple sclerosis)*	2 (6.1%)	1 (4.2%)	15 (17.6%)	0.128
	Gastrointestinal diseases (such as inflammatory bowel disease)*	3 (9.1%)	3 (12.5%)	26 (30.6%)	0.019
	Kidney diseases (such as chronic kidney disease)	7 (21.2%)	3 (12.5%)	19 (22.4%)	0.643
	Liver diseases (such as cirrhosis)	7 (21.2%)	6 (25.0%)	18 (21.2%)	0.92
	Mental health disorders (including depression, anxiety, and dementia)	6 (18.2%)	5 (20.8%)	21 (24.7%)	0.784
	Cancer	2 (6.1%)	0 (0.0%)	8 (9.4%)	0.338
	I’m not sure	11 (33.3%)	2 (8.3%)	13 (15.3%)	0.029
How do you rate the quality of existing scientific research on the efficacy and safety of Ayurveda in managing chronic conditions?	High quality	0 (0.0%)	3 (12.5%)	4 (4.7%)	< 0.001
	Moderate quality	0 (0.0%)	7 (29.2%)	8 (9.4%)	
	Low quality	17 (51.5%)	10 (41.7%)	14 (16.5%)	
	Not sure	16 (48.5%)	4 (16.7%)	59 (69.4%)	
How much do you agree with the statement ‘I’m equipped enough to counsel patients on the benefits and harms of Ayurvedic interventions in chronic condition management?	Strongly agree	0 (0.0%)	0 (0.0%)	6 (7.1%)	< 0.001
	Agree	0 (0.0%)	3 (12.5%)	5 (5.9%)	
	Neutral	0 (0.0%)	6 (25.0%)	7 (8.2%)	
	Disagree	17 (51.5%)	11 (45.8%)	16 (18.8%)	
	Strongly disagree	16 (48.5%)	4 (16.7%)	51 (60.0%)	
How easy is it for you to find reliable information regarding Ayurveda?	Very easy	0 (0.0%)	2 (8.3%)	5 (5.9%)	< 0.001
	Easy	0 (0.0%)	3 (12.5%)	5 (5.9%)	
	Neither difficult nor easy	1 (3.0%)	2 (8.3%)	8 (9.4%)	
	Difficult	17 (51.5%)	11 (45.8%)	14 (16.5%)	
	Very difficult	3 (9.1%)	4 (16.7%)	6 (7.1%)	
	I don’t know how to access this information	12 (36.4%)	2 (8.3%)	47 (55.3%)	
What sources do you typically use to obtain information about Ayurveda?	Medical journals	20 (60.6%)	13 (54.2%)	45 (52.9%)	0.752
	Conferences or seminars	2 (6.1%)	3 (12.5%)	4 (4.7%)	0.344
	Online resources	4 (12.1%)	5 (20.8%)	26 (30.6%)	0.101
	Colleagues or peers (websites, blogs, forums, etc.)	5 (15.2%)	3 (12.5%)	15 (17.6%)	0.902
Have you ever undergone training in Ayurveda or participated in any Ayurveda courses or workshops?	Yes, extensively	0 (0.0%)	5 (20.8%)	3 (3.5%)	< 0.001
	Yes, moderately	0 (0.0%)	4 (16.7%)	2 (2.4%)	
	Yes, minimally	0 (0.0%)	3 (12.5%)	4 (4.7%)	
	No	33 (100%)	12 (50.0%)	76 (89.4%)	
How familiar are you with the legal and regulatory issues related to Ayurveda?	Very familiar	0 (0.0%)	5 (20.8%)	1 (1.2%)	< 0.001
	Somewhat familiar	0 (0.0%)	4 (16.7%)	5 (5.9%)	
	Not very familiar	17 (51.5%)	11 (45.8%)	9 (10.6%)	
	Not at all familiar	16 (48.5%)	4 (16.7%)	70 (82.4%)	

Association between ethnicity and attitude of UK HCPs on Ayurveda for chronic condition management					
**Variable**	**Attributes (n)**	**Caucasian** **(33)**	**Black** **(24)**	**Asian** **(85)**	**p-value**
Ayurveda should be included in the treatment options offered to patients with chronic conditions	Strongly agree	1 (3.0%)	4 (16.7%)	12 (14.1%)	< 0.001
	Agree	19 (57.6%)	12 (50.0%)	16 (18.8%)	
	Neutral	10 (30.3%)	7 (29.2%)	50 (58.8%)	
	Disagree	1 (3.0%)	0 (0.0%)	5 (5.9%)	
	Strongly disagree	2 (6.1%)	1 (4.2%)	2 (2.4%)	
I believe that healthcare professionals should discuss about Ayurveda with their patients	Strongly agree	2 (6.1%)	5 (20.8%)	12 (14.1%)	0.009
	Agree	6 (18.2%)	12 (50.0%)	38 (44.7%)	
	Neutral	21 (63.6%)	6 (25.0%)	26 (30.6%)	
	Disagree	2 (6.1%)	1 (4.2%)	8 (9.4%)	
	Strongly disagree	2 (6.1%)	0 (0.0%)	1 (1.2%)	
Ayurveda should be accessible via NHS for people living with chronic conditions in the UK	Strongly agree	1 (3.0%)	2 (8.3%)	8 (9.4%)	0.001
	Agree	18 (54.5%)	14 (58.3%)	20 (23.5%)	
	Neutral	11 (33.3%)	6 (25.0%)	51 (60.0%)	
	Disagree	0 (0.0%)	2 (8.3%)	4 (4.7%)	
	Strongly disagree	3 (9.1%)	0 (0.0%)	2 (2.4%)	
The usage of ayurvedic interventions should be regulated by MHRA (Medicines and Healthcare products Regulatory Agency)/ NHS in the UK	Strongly agree	23 (69.7%)	14 (58.3%)	18 (21.2%)	< 0.001
	Agree	6 (18.2%)	2 (8.3%)	55 (64.7%)	
	Neutral	3 (9.1%)	3 (12.5%)	10 (11.8%)	
	Disagree	0 (0.0%)	2 (8.3%)	2 (2.4%)	
	Strongly disagree	1 (3.0%)	3 (12.5%)	0 (0.0%)	
The lack of regulation in the practice of Ayurveda poses a risk to patients with chronic conditions	Strongly agree	24 (72.7%)	12 (50.0%)	18 (21.2%)	< 0.001
	Agree	7 (21.2%)	4 (16.7%)	56 (65.9%)	
	Neutral	2 (6.1%)	4 (16.7%)	7 (8.2%)	
	Disagree	0 (0.0%)	3 (12.5%)	4 (4.7%)	
	Strongly disagree	0 (0.0%)	1 (4.2%)	0 (0.0%)	
Healthcare professionals should work collaboratively with Ayurvedic practitioners to provide the best care for patients with chronic conditions	Strongly agree	7 (21.2%)	12 (50.0%)	15 (17.6%)	< 0.001
	Agree	5 (15.2%)	5 (20.8%)	56 (65.9%)	
	Neutral	18 (54.5%)	3 (12.5%)	9 (10.6%)	
	Disagree	1 (3.0%)	2 (8.3%)	3 (3.5%)	
	Strongly disagree	2 (6.1%)	2 (8.3%)	2 (2.4%)	
I would be willing to collaborate with Ayurvedic practitioners in managing patients with chronic conditions	Strongly agree	1 (3.0%)	4 (16.7%)	15 (17.6%)	< 0.001
	Agree	20 (60.6%)	13 (54.2%)	13 (15.3%)	
	Neutral	10 (30.3%)	3 (12.5%)	53 (62.4%)	
	Disagree	0 (0.0%)	3 (12.5%)	3 (3.5%)	
	Strongly disagree	2 (6.1%)	1 (4.2%)	1 (1.2%)	
I would be willing to refer patients for Ayurvedic treatment if it were available and appropriate	Strongly agree	1 (3.0%)	4 (16.7%)	14 (16.5%)	< 0.001
	Agree	4 (12.1%)	5 (20.8%)	57 (67.1%)	
	Neutral	24 (72.7%)	11 (45.8%)	7 (8.2%)	
	Disagree	2 (6.1%)	4 (16.7%)	5 (5.9%)	
	Strongly disagree	2 (6.1%)	0 (0.0%)	2 (2.4%)	
There should be a formal referral system in place between conventional healthcare professionals and Ayurvedic practitioners	Strongly agree	2 (6.1%)	4 (16.7%)	13 (15.3%)	< 0.001
	Agree	17 (51.5%)	13 (54.2%)	16 (18.8%)	
	Neutral	10 (30.3%)	3 (12.5%)	48 (56.5%)	
	Disagree	2 (6.1%)	2 (8.3%)	6 (7.1%)	
	Strongly disagree	2 (6.1%)	2 (8.3%)	2 (2.4%)	
The referral system to an Ayurvedic practitioner should be regulated by the government in UK	Strongly agree	6 (18.2%)	3 (12.5%)	56 (65.9%)	< 0.001
	Agree	21 (63.6%)	12 (50.0%)	20 (23.5%)	
	Neutral	5 (15.2%)	3 (12.5%)	6 (7.1%)	
	Disagree	0 (0.0%)	4 (16.7%)	1 (1.2%)	
	Strongly disagree	1 (3.0%)	2 (8.3%)	2 (2.4%)	
Ayurvedic interventions should be funded via the National Health Service (NHS) in the UK	Strongly agree	1 (3.0%)	2 (8.3%)	8 (9.4%)	< 0.001
	Agree	17 (51.5%)	13 (54.2%)	15 (17.6%)	
	Neutral	9 (27.3%)	3 (12.5%)	57 (67.1%)	
	Disagree	4 (12.1%)	4 (16.7%)	3 (3.5%)	
	Strongly disagree	2 (6.1%)	2 (8.3%)	2 (2.4%)	
Patients who use Ayurveda for the management of chronic conditions should have access to qualified and trained practitioners	Strongly agree	22 (66.7%)	12 (50.0%)	20 (23.5%)	< 0.001
	Agree	7 (21.2%)	2 (8.3%)	58 (68.2%)	
	Neutral	2 (6.1%)	6 (25.0%)	6 (7.1%)	
	Disagree	1 (3.0%)	4 (16.7%)	1 (1.2%)	
	Strongly disagree	1 (3.0%)	0 (0.0%)	0 (0.0%)	
I believe that Ayurveda can be used to prevent the onset of chronic conditions	Strongly agree	5 (15.2%)	9 (37.5%)	14 (16.5%)	< 0.001
	Agree	12 (36.4%)	8 (33.3%)	12 (14.1%)	
	Neutral	13 (39.4%)	3 (12.5%)	55 (64.7%)	
	Disagree	0 (0.0%)	4 (16.7%)	3 (3.5%)	
	Strongly disagree	3 (9.1%)	0 (0.0%)	1 (1.2%)	
I believe Ayurveda can help manage chronic health conditions	Strongly agree	0 (0.0%)	1 (4.2%)	15 (17.6%)	< 0.001
	Agree	19 (57.6%)	13 (54.2%)	16 (18.8%)	
	Neutral	11 (33.3%)	5 (20.8%)	52 (61.2%)	
	Disagree	2 (6.1%)	3 (12.5%)	2 (2.4%)	
	Strongly disagree	1 (3.0%)	2 (8.3%)	0 (0.0%)	
Ayurveda can be used to address the root cause of chronic conditions rather than just treating the symptoms	Strongly agree	6 (18.2%)	9 (37.5%)	12 (14.1%)	< 0.001
	Agree	12 (36.4%)	2 (8.3%)	13 (15.3%)	
	Neutral	11 (33.3%)	7 (29.2%)	56 (65.9%)	
	Disagree	1 (3.0%)	4 (16.7%)	2 (2.4%)	
	Strongly disagree	3 (9.1%)	2 (8.3%)	2 (2.4%)	
I believe that Ayurveda has the potential to improve the quality of life of patients with chronic conditions	Strongly agree	2 (6.1%)	2 (8.3%)	14 (16.5%)	< 0.001
	Agree	18 (54.5%)	12 (50.0%)	18 (21.2%)	
	Neutral	11 (33.3%)	4 (16.7%)	50 (58.8%)	
	Disagree	0 (0.0%)	2 (8.3%)	3 (3.5%)	
	Strongly disagree	2 (6.1%)	4 (16.7%)	0 (0.0%)	
I think that Ayurveda has the potential to help patients with chronic conditions who have not been helped by conventional medicine	Strongly agree	0 (0.0%)	3 (12.5%)	14 (16.5%)	0.001
	Agree	19 (57.6%)	10 (41.7%)	21 (24.7%)	
	Neutral	13 (39.4%)	7 (29.2%)	47 (55.3%)	
	Disagree	0 (0.0%)	2 (8.3%)	1 (1.2%)	
	Strongly disagree	1 (3.0%)	2 (8.3%)	2 (2.4%)	
Incorporation of Ayurvedic therapies into routine clinical practice would result in increased patient satisfaction	Strongly agree	1 (3.0%)	3 (12.5%)	11 (12.9%)	< 0.001
	Agree	20 (60.6%)	10 (41.7%)	17 (20.0%)	
	Neutral	9 (27.3%)	7 (29.2%)	54 (63.5%)	
	Disagree	0 (0.0%)	4 (16.7%)	3 (3.5%)	
	Strongly disagree	3 (9.1%)	0 (0.0%)	0 (0.0%)	
I believe that Ayurveda can contribute to patient-centred care	Strongly agree	2 (6.1%)	3 (12.5%)	13 (15.3%)	< 0.001
	Agree	5 (15.2%)	4 (16.7%)	60 (70.6%)	
	Neutral	24 (72.7%)	13 (54.2%)	9 (10.6%)	
	Disagree	2 (6.1%)	3 (12.5%)	3 (3.5%)	
	Strongly disagree	0 (0.0%)	1 (4.2%)	0 (0.0%)	
There should be more research into the efficacy and safety of Ayurveda for the management of chronic conditions	Strongly agree	9 (27.3%)	4 (16.7%)	66 (77.6%)	< 0.001
	Agree	19 (57.6%)	11 (45.8%)	14 (16.5%)	
	Neutral	4 (12.1%)	3 (12.5%)	1 (1.2%)	
	Disagree	1 (3.0%)	1 (4.2%)	3 (3.5%)	
	Strongly disagree	0 (0.0%)	5 (20.8%)	1 (1.2%)	
More resources should be allocated to research the potential benefits of Ayurveda for the management of chronic conditions	Strongly agree	3 (9.1%)	4 (16.7%)	60 (70.6%)	< 0.001
	Agree	21 (63.6%)	10 (41.7%)	14 (16.5%)	
	Neutral	6 (18.2%)	7 (29.2%)	6 (7.1%)	
	Disagree	1 (3.0%)	3 (12.5%)	5 (5.9%)	
	Strongly disagree	2 (6.1%)	0 (0.0%)	0 (0.0%)	
Healthcare professionals should work with Ayurvedic practitioners to develop evidence-based guidelines for the management of chronic conditions	Strongly agree	20 (60.6%)	15 (62.5%)	15 (17.6%)	< 0.001
	Agree	5 (15.2%)	3 (12.5%)	57 (67.1%)	
	Neutral	5 (15.2%)	1 (4.2%)	8 (9.4%)	
	Disagree	1 (3.0%)	4 (16.7%)	4 (4.7%)	
	Strongly disagree	2 (6.1%)	1 (4.2%)	1 (1.2%)	
In your opinion, what should be done to ensure the safe and effective use of Ayurveda in the UK, assuming a consensus among the public	Develop guidelines and regulations	14 (42.4%)	4 (16.7%)	36 (42.4%)	0.061
	Educate healthcare professionals and patients	12 (36.4%)	7 (29.2%)	43 (50.6%)	0.11
	Allocate more resources in facilitating the use of Ayurveda	8 (24.2%)	4 (16.7%)	24 (28.2%)	0.509
	Collaborate with Ayurvedic practitioners and organizations to promote greater transparency and accountability in the industry	12 (36.4%)	9 (37.5%)	21 (24.7%)	0.298
	Conduct more research on Ayurvedic interventions	17 (51.5%)	8 (33.3%)	32 (37.6%)	0.292
Patients should be informed of both the benefits and risks of using Ayurveda as a treatment option	Strongly agree	12 (36.4%)	12 (50.0%)	42 (49.4%)	< 0.001
	Agree	17 (51.5%)	1 (4.2%)	36 (42.4%)	
	Neutral	3 (9.1%)	3 (12.5%)	5 (5.9%)	
	Disagree	0 (0.0%)	6 (25.0%)	2 (2.4%)	
	Strongly disagree	1 (3.0%)	2 (8.3%)	0 (0.0%)	
I’m willing to learn more about the potential role of Ayurveda in chronic conditions management	Strongly agree	8 (24.2%)	3 (12.5%)	60 (70.6%)	< 0.001
	Agree	21 (63.6%)	15 (62.5%)	17 (20.0%)	
	Neutral	2 (6.1%)	2 (8.3%)	5 (5.9%)	
	Disagree	0 (0.0%)	1 (4.2%)	1 (1.2%)	
	Strongly disagree	2 (6.1%)	3 (12.5%)	2 (2.4%)	
I believe that orientation to Ayurveda through educational training should be offered to healthcare professionals	Strongly agree	21 (63.6%)	13 (54.2%)	16 (18.8%)	< 0.001
	Agree	7 (21.2%)	2 (8.3%)	59 (69.4%)	
	Neutral	2 (6.1%)	5 (20.8%)	8 (9.4%)	
	Disagree	1 (3.0%)	2 (8.3%)	2 (2.4%)	
	Strongly disagree	2 (6.1%)	2 (8.3%)	0 (0.0%)	

Association between ethnicity and training needs of UK HCPs’ on Ayurveda in chronic conditions management					
**Variable**	**Attributes (n)**	**Caucasian** **(33)**	**Black** **(24)**	**Asian** **(85)**	**p-value**
How often do you perceive interest from patients in discussing Ayurvedic interventions with you	Frequently	0 (0.0%)	8 (33.3%)	1 (1.2%)	< 0.001
	Occasionally	2 (6.1%)	1 (4.2%)	18 (21.2%)	
	Rarely	3 (9.1%)	3 (12.5%)	58 (68.2%)	
	Never	28 (84.8%)	12 (50.0%)	8 (9.4%)	
If a patient expresses interest in using Ayurveda, how do you typically respond	Provide information	17 (51.5%)	9 (37.5%)	8 (9.4%)	< 0.001
	Refer the patient to an Ayurvedic practitioner	0 (0.0%)	4 (16.7%)	7 (8.2%)	
	Offer to work with an Ayurvedic practitioner to provide care for the patient	1 (3.0%)	4 (16.7%)	3 (3.5%)	
	Discouraged them from seeking Ayurvedic treatment	0 (0.0%)	3 (12.5%)	2 (2.4%)	
	Don’t offer any definitive advice	8 (24.2%)	1 (4.2%)	59 (69.4%)	
	Other	7 (21.2%)	3 (12.5%)	6 (7.1%)	
In your professional capacity, how comfortable are you in communicating with your patients regarding Ayurvedic interventions?	Extremely comfortable	0 (0.0%)	4 (16.7%)	2 (2.4%)	< 0.001
	Moderately comfortable	1 (3.0%)	7 (29.2%)	7 (8.2%)	
	Slightly comfortable	18 (54.5%)	11 (45.8%)	12 (14.1%)	
	Not at all comfortable	14 (42.4%)	2 (8.3%)	64 (75.3%)	
With approximately what percentage of your patients do you talk about possible benefits of using a Ayurvedic treatment ?	0%	15 (45.5%)	4 (16.7%)	62 (72.9%)	< 0.001
	1–25%	18 (54.5%)	8 (33.3%)	16 (18.8%)	
	26–50%	0 (0.0%)	5 (20.8%)	5 (5.9%)	
	51–75%	0 (0.0%)	6 (25.0%)	2 (2.4%)	
	76–100%	0 (0.0%)	1 (4.2%)	0 (0.0%)	
With approximately what percentage of your patients do you talk about possible harmful outcomes of using Ayurvedic interventions	0%	16 (48.5%)	4 (16.7%)	69 (81.2%)	< 0.001
	1–25%	17 (51.5%)	9 (37.5%)	8 (9.4%)	
	26–50%	0 (0.0%)	4 (16.7%)	4 (4.7%)	
	51–75%	0 (0.0%)	6 (25.0%)	4 (4.7%)	
	76–100%	0 (0.0%)	1 (4.2%)	0 (0.0%)	
Who usually initiates discussions of benefits and risks of a Ayurvedic intervention?	I initiate the discussion	0 (0.0%)	2 (8.3%)	4 (4.7%)	< 0.001
	Patient initiates the discussion	20 (60.6%)	10 (41.7%)	17 (20.0%)	
	Sometimes I and sometimes the patient initiates the discussion	0 (0.0%)	3 (12.5%)	8 (9.4%)	
	Third party initiates the discussion	0 (0.0%)	5 (20.8%)	2 (2.4%)	
	Not Applicable	13 (39.4%)	4 (16.7%)	54 (63.5%)	
Do you routinely ask patients whether or not they are using Ayurvedic interventions as part of their healthcare?	Yes, always	0 (0.0%)	3 (12.5%)	2 (2.4%)	< 0.001
	Yes, sometimes	17 (51.5%)	13 (54.2%)	12 (14.1%)	
	No, rarely	2 (6.1%)	4 (16.7%)	11 (12.9%)	
	No, never	11 (33.3%)	3 (12.5%)	53 (62.4%)	
	Not applicable to my practice	3 (9.1%)	1 (4.2%)	7 (8.2%)	
How often do you think patients report using Ayurveda without disclosing it to you?	Frequently	1 (3.0%)	5 (20.8%)	5 (5.9%)	< 0.001
	Occasionally	7 (21.2%)	4 (16.7%)	58 (68.2%)	
	Rarely	20 (60.6%)	10 (41.7%)	14 (16.5%)	
	Never	5 (15.2%)	5 (20.8%)	8 (9.4%)	
How likely is it that you would refer a patient to an Ayurvedic practitioner for treatment of a chronic condition?	Extremely likely	0 (0.0%)	1 (4.2%)	5 (5.9%)	< 0.001
	Somewhat likely	1 (3.0%)	3 (12.5%)	7 (8.2%)	
	Neither Likely Nor Unlikely	21 (63.6%)	9 (37.5%)	14 (16.5%)	
	Somewhat Unlikely	2 (6.1%)	8 (33.3%)	9 (10.6%)	
	Extremely Unlikely	9 (27.3%)	3 (12.5%)	50 (58.8%)	
Have you ever referred a patient to an Ayurvedic practitioner?	Yes	0 (0.0%)	5 (20.8%)	6 (7.1%)	0.012
	No	33 (100%)	19 (79.2%)	79 (92.9%)	
If yes, was your reason for referral?	The patient expressed an interest in Ayurvedic interventions	0 (0.0%)	8 (33.3%)	3 (3.5%)	< 0.001
	Ayurvedic interventions were recommended as a complement to allopathic treatments	16 (48.5%)	10 (41.7%)	2 (2.4%)	
	The patient had a chronic condition that was not responding well to allopathic treatments	0 (0.0%)	2 (8.3%)	42 (49.4%)	
	Other	17 (51.5%)	4 (16.7%)	38 (44.7%)	
If no, what has stopped you from referring patients to an Ayurvedic practitioner?	Lack of knowledge	7 (21.2%)	3 (12.5%)	57 (67.1%)	< 0.001
	Concerns about safety	0 (0.0%)	5 (20.8%)	7 (8.2%)	
	Lack of evidence for efficacy	2 (6.1%)	4 (16.7%)	6 (7.1%)	
	Cost	0 (0.0%)	3 (12.5%)	0 (0.0%)	
	Lack of access to Ayurvedic practitioners	18 (54.5%)	9 (37.5%)	11 (12.9%)	
With approximately what percentage of your patients do you talk about possible harmful outcomes of using Ayurvedic interventions	0%	16 (48.5%)	4 (16.7%)	69 (81.2%)	< 0.001
	1–25%	17 (51.5%)	9 (37.5%)	8 (9.4%)	
	26–50%	0 (0.0%)	4 (16.7%)	4 (4.7%)	
	51–75%	0 (0.0%)	6 (25.0%)	4 (4.7%)	
	76–100%	0 (0.0%)	1 (4.2%)	0 (0.0%)	
I have observed positive outcomes with Ayurvedic treatment. (personal experience or on the experiences of patients)	Yes	20 (60.6%)	15 (62.5%)	24 (28.2%)	< 0.001
	No	13 (39.4%)	9 (37.5%)	61 (71.8%)	
How often do you discuss Ayurveda with other healthcare professionals (e.g. colleagues, specialists)?	Frequently	0 (0.0%)	5 (20.8%)	6 (7.1%)	< 0.001
	Occasionally	0 (0.0%)	7 (29.2%)	10 (11.8%)	
	Rarely	18 (54.5%)	10 (41.7%)	11 (12.9%)	
	Never	15 (45.5%)	2 (8.3%)	58 (68.2%)	
How often do you perceive conflict between Ayurveda and conventional medicine paradigms?	Frequently	1 (3.0%)	2 (8.3%)	12 (14.1%)	< 0.001
	Occasionally	3 (9.1%)	5 (20.8%)	42 (49.4%)	
	Rarely	3 (9.1%)	5 (20.8%)	12 (14.1%)	
	Never	26 (78.8%)	12 (50.0%)	19 (22.4%)	
How often do you perceive lack of acceptance from colleagues regarding use of Ayurveda for chronic condition management?	Frequently	5 (15.2%)	7 (29.2%)	58 (68.2%)	< 0.001
	Occasionally	2 (6.1%)	3 (12.5%)	13 (15.3%)	
	Rarely	1 (3.0%)	4 (16.7%)	8 (9.4%)	
	Never	25 (75.8%)	10 (41.7%)	6 (7.1%)	
Would you be willing to collaborate with Ayurvedic practitioners in patient care	Yes	26 (78.8%)	18 (75.0%)	76 (89.4%)	< 0.001
	No	7 (21.2%)	6 (25.0%)	9 (10.6%)	
What do you perceive as the biggest challenge to using Ayurveda for chronic conditions in the UK?	Lack of scientific evidence	10 (30.3%)	1 (4.2%)	67 (78.8%)	< 0.001
	Lack of regulatory framework	7 (21.2%)	4 (16.7%)	24 (28.2%)	0.444
	Limited understanding of Ayurveda among conventional healthcare professionals	25 (75.8%)	9 (37.5%)	29 (34.1%)	< 0.001
	Limited patient demand	5 (15.2%)	6 (25.0%)	15 (17.6%)	0.618
	Limited resources	6 (18.2%)	3 (12.5%)	18 (21.2%)	0.627
Do you think the current regulatory framework is sufficient to ensure patient safety?	Yes	4 (12.1%)	10 (41.7%)	10 (11.8%)	0.004
	No	29 (87.9%)	14 (58.3%)	75 (88.2%)	
Do you think there are enough resources available to support the use of Ayurveda into healthcare in the UK?	Yes	3 (9.1%)	5 (20.8%)	5 (5.9%)	0.093
	No	30 (90.9%)	19 (79.2%)	80 (94.1%)	
What steps do you think can be taken to overcome the challenges to using Ayurveda in the UK?	Increase scientific research on Ayurveda	16 (48.5%)	8 (33.3%)	48 (56.5%)	0.129
	Develop a regulatory framework for Ayurveda	15 (45.5%)	9 (37.5%)	41 (48.2%)	0.647
	Provide education and training to conventional healthcare professionals	17 (51.5%)	8 (33.3%)	43 (50.6%)	0.292
	Increase awareness among patients	6 (18.2%)	3 (12.5%)	32 (37.6%)	0.017
	Allocate more resources	3 (9.1%)	5 (20.8%)	34 (40.0%)	0.003

*p*-values were calculated using Chi-Square tests except where expected cell counts were < 5, in which case Fisher’s Exact Tests were used

#### Association between ethnicity and attitude of UK HCPs on ayurveda for chronic condition management

The data showed a statistically significant association (*p* < 0.001) between ethnicity and the attitude of UK HCPs on Ayurveda for chronic condition management. Black participants showed the highest support for including Ayurveda in treatment options (16.7% strongly agree, 50% agree), with majority (50% strongly agree, 20.8% agree) advocating for collaboration with Ayurvedic practitioners and a similar percentage (16.7% strongly agree, 54.2% agree) supporting a formal referral system. They also demonstrated strong confidence in Ayurveda’s preventive role for chronic conditions (37.5% strongly agree, 33.3% agree). In contrast, Caucasian participants expressed the strongest support for regulating Ayurvedic interventions (69.7% strongly agree, 18.2% agree), and also exhibited strong need for the accessibility of qualified practitioners (66.7% strongly agree, 21.2% agree). They reported moderate beliefs regarding Ayurveda’s effectiveness in managing chronic conditions (57.6%), with notable uncertainty reflected in high neutral responses.

Asian participants demonstrated the highest willingness to personally refer patients for Ayurvedic interventions (16.5% strongly agree, 67.1% agree) and overwhelming support for government regulation of referral systems (65.9% strongly agree, 23.5% agree). They expressed significant ambivalence regarding Ayurveda’s effectiveness, with only 36.4% (17.6% strongly agree, 18.8% agree) believing in its ability to manage chronic conditions, and 61.2% remaining neutral. Additionally, Asian participants strongly advocated for increased research on Ayurveda’s efficacy in chronic condition management (77.6% strongly agree, 16.5% agree). They overwhelmingly supported patient education (49.4% strongly agree, 42.4% agree) and expressed a desire to learn more (70.6% strongly agree, 20% agree). Overall, while collaboration between HCPs and Ayurvedic practitioners received broad support across all groups, Black and Caucasian participants showed stronger inclinations toward developing evidence-based guidelines. (Table 2).

#### Association between ethnicity and UK hcps’ training needs on ayurveda

The survey revealed a statistically significant association (*p* < 0.001) between ethnicity and the UK HCPs’ training needs on Ayurveda. Black participants reported the highest patient interest in discussing Ayurvedic interventions (33.3% frequently) and expressed greater comfort in these discussions (16.7% extremely comfortable and 29.2% moderately comfortable). In contrast, Caucasian participants noted less interest (84.8% never) and primarily rely on patients to initiate discussions (60.6%). Asian participants showed the lowest in discussing Ayurveda, with majority (75.3%) feeling “not at all comfortable” discussing Ayurveda.

While Black professionals are more likely to refer (4.2% extremely likely, 12.5% likely), Asian participants demonstrated the most hesitancy with majority (58.8%) extremely unlikely to refer. Reasons for not referring included lack of access to Ayurvedic practitioners for Caucasian participants (54.5%), safety concerns for Black (20.8%), and lack of knowledge for Asian participants (67.1%). Despite these hesitations, a majority of Caucasian (60.6%) and Black (62.5%) participants observed positive outcomes from Ayurveda, compared to only 28.2% of Asian participants. Asian participants felt the greatest perceived conflict with conventional medicine (14.1% frequently, 49.4% occasionally) and a greater perceived lack of acceptance from colleagues regarding Ayurveda in chronic condition management (68.2% frequently, 15.3% occasionally). (Table 2).

## Discussion

This is the first UK study examining UK healthcare professionals’ awareness, attitudes, and knowledge gaps regarding Ayurveda in chronic disease management. Our findings reveal mixed views on awareness and attitudes but a clear consensus on the need for additional training. These results mirror existing literature on CAM [8, 13–16, 23, 24], highlighting global challenges in discussing traditional health systems with patients, particularly due to safety concerns, limited knowledge, and the lack of robust clinical evidence [13–15, 19, 20, 23]. 

Awareness of Ayurveda among UK HCPs: We found a moderate level of familiarity with Ayurveda among UK HCPs, with awareness skewed toward herbal and dietary interventions and lower for more advanced practices such as Panchakarma therapies and para-surgical interventions. Limited understanding of Ayurveda’s efficacy and safety was also evident, with nearly three-quarters of participants acknowledging minimal or no understanding, findings consistent with international CAM research [8, 13–16, 23, 24]. This baseline awareness, while not equivalent to formal expertise, underscores the need to identify knowledge gaps and address training shortfalls. The absence of formal education on traditional systems such as Ayurveda within the UK medical curriculum makes this assessment particularly important in a country serving a large South Asian population.

Attitudes toward the Role of Ayurveda in Chronic Disease Management: A largemajority of HCPs agreed that patients using Ayurveda should have access to qualified and trained practitioners, emphasising the importance of professional expertise. Beliefs about Ayurveda’s role were mixed, respondents tended to see it as more beneficial for prevention than for treatment of established conditions. Yet many acknowledged its potential to improve patients’ quality of life and benefit those who do not respond to conventional treatments. Despite this, support for integrating Ayurvedic therapies into routine practice remained moderate, with many HCPs expressing neutrality. However, there was strong support for increased research, resource allocation, and the development of evidence-based guidelines through collaboration between HCPs and Ayurvedic practitioners.

These findings echo international studies showing positive attitudes toward CAM. Senior pharmacy students in Andhra Pradesh, UK physicians in palliative care, Kuwaiti medical and pharmacy students, Irish medical students, and Australian medical interns have all reported supportive attitudes toward CAM’s integration into healthcare, particularly when evidence and training are provided. This pattern underscores a global trend toward validating CAM approaches and building structured pathways for integration.

Attitudes toward Education and Training in Ayurveda: Our study highlights a strong endorsement for educating patients about Ayurveda and reflects a commitment to informed decision-making and the promotion of safe practices. High interest in expanding personal knowledge underscores HCPs’ openness to integrative approaches. These findings are consistent with literature from multiple countries: allopathic resident doctors in India and Turkish academicians support including CAM in curricula; UK academic doctors’ express interest in CAM training; Australian medical interns emphasise its importance for junior doctors; and similar sentiments are echoed among Iranian psychologists and German palliative care professionals. Together, these findings advocate for integrating CAM education into healthcare training, albeit with voluntary or elective components to respect diverse perspectives. Addressing these educational gaps through structured curricula and targeted training programs is crucial for safe and effective integration of Ayurveda into modern healthcare.

UK HCPs’ Practices with Ayurvedic Medicine in Chronic Disease Management: Despite moderate awareness and openness, a majority of HCPs in the UK rarely or never perceive patient interest in discussing Ayurvedic interventions, and when interest is expressed, nearly half offer no definitive advice. Many HCPs feel uncomfortable discussing Ayurveda, with few addressing its potential benefits or risks. This hesitancy likely stems from inadequate training, uncertainty about efficacy and safety, and the absence of formal education on CAM. Patient non-disclosure is also common and mirrors international findings: studies in Malaysia, South India, Pakistan, Saudi Arabia, and the UAE show many patients conceal CAM use from their doctors due to fear of negative reactions or because practitioners did not ask. When credible information from HCPs is lacking, patients often turn to non-medical sources, such as advertisements or personal recommendations, creating potential safety risks. This underscores the need for HCPs to be well-informed about CAM to ensure patient safety, manage drug-herb interactions, and maintain trust in the patient–clinician relationship.

Factors Influencing Scepticism and Integration: Scepticism among Caucasian HCPs may be rooted in legitimate factors, including variability in Ayurvedic research quality, a paucity of large-scale rigorous clinical trials, and cultural differences in conceptualising health and wellness. Recognising these nuances is essential for framing constructive dialogue and designing future research and educational initiatives that bridge conventional and traditional medicine.

Insufficient regulation of Ayurveda is another frequently cited barrier to integration, but it may equally reflect legitimate patient-safety concerns. Without consistent quality control, evidence standards, and practitioner accreditation, unregulated use of Ayurvedic products or practices could pose risks. Thus, regulatory frameworks should not only facilitate integration but also safeguard patient safety and public trust.

Implications for Policy, Research, and Practice: A key finding is the need for targeted training to facilitate effective patient-HCP dialogues about Ayurveda in clinical settings. In increasingly diverse populations, such as the UK, chronic condition management often intersects with cultural health practices. Yet, most HCPs are not adequately prepared during undergraduate or postgraduate training to address these preferences, potentially leading to missed opportunities for shared decision-making. This gap is especially concerning given evidence of poor medication adherence among minority ethnic groups, partly driven by interest in alternative remedies and health beliefs favouring non-pharmacological approaches. Without proper training, HCPs may struggle to address these preferences, further hindering shared clinical decision-making.

Our findings underscore the importance of research and regulation, with over half of participants advocating for high-quality studies to validate Ayurveda’s efficacy and many emphasising the need for a robust regulatory framework. Addressing scepticism and perceived irrelevance requires condition-specific evidence and collaborative care models. Strong support emerged for partnerships between HCPs and Ayurvedic practitioners, aligning with patient-centred care principles. Formal referral systems and collaborative frameworks could facilitate the safe and effective integration of Ayurveda into mainstream healthcare.

## Limitations

The developed questionnaire was effective in understanding the awareness of HCPs and also identifying training gaps with a specific focus on Ayurveda. However, the study did not explore underlying attitudes or barriers to integration, limiting a comprehensive understanding. The number of responses were low, potentially due to timing, survey fatigue, niche interest, and possible response bias, hence affecting generalisability. The respondent demographic, predominantly mid-to-late career professionals from secondary care in Yorkshire and the Humber, may limit the broader applicability of the results. The sample had a high proportion of Asian/Asian British participants, which, while reflective of certain communities with cultural ties to Ayurveda, may skew the overall awareness and attitudes reported. Despite these issues, the study offers valuable insights to inform future training programs for HCPs.

## Conclusion

The findings from this survey highlight the need to open dialogues about traditional systems of medicine like Ayurveda, while also acknowledging the challenges in initiating such discussions. To properly address the needs of a diverse patient population, HCPs require enhanced training on Ayurvedic practices to improve their confidence and ability to discuss treatment options with patients. Incorporating comprehensive Ayurveda education into medical curricula and providing ongoing professional development are essential steps. The results of this study have informed the development of an initial educational video for HCPs, serving as a practical resource to address identified knowledge gaps and support informed decision-making regarding the use of Ayurveda in clinical practice.

## Supplementary Information

Supplementary Material 1.

## Data Availability

The datasets generated and/or analysed during the current study are available from the corresponding author on reasonable request.

## Abbreviations

CAM: Complementary and Alternative Medicine
HCPs: Healthcare Professionals
MHRA: Medicines and Healthcare products Regulatory Agency
NHS: National Health Service
STROBE: Strengthening the Reporting of Observational Studies in Epidemiology
