# Supplementary material for: Awareness and attitudes towards ayurveda in chronic disease management and related knowledge gaps among UK healthcare professionals: an analytical cross-sectional survey
Source: BMC Complement Med Ther. 2026 Mar 6;26:89. doi: 10.1186/s12906-026-05283-9 (PMC12967011; doi:10.1186/s12906-026-05283-9)
Supplement: Supplementary file 1 — Supplementary Material 1. [file 12906_2026_5283_MOESM1_ESM.docx]

Supplementary material: Questionnaire for the survey on Awareness, attitudes of Ayurveda and training needs of the UK healthcare professionals in the management of chronic conditions

**Domain 1: Demographic details**

1. Age:

- 18-24
- 25-34
- 35-44
- 45-54
- 55-64
- 65-74
- 75 and older

1. Gender:

- Male
- Female
- Non-binary
- Prefer not to say

1. Ethnicity:

- White
- Black/Black British/African/Caribbean
- Asian or Asian British
- Mixed or Multiple ethnic groups
- Other ethnic group
- Prefer not to say

1. Geographic location:

- North East
- North West
- Yorkshire and the Humber
- East Midlands
- West Midlands
- East of England
- London
- South East
- South West
- Scotland
- Wales
- Northern Ireland

1. Profession:

- Medical specialty doctor
- General practitioner
- Nurse

1. If you selected "Medical specialty doctor" in the previous question, please specify your specialty: ________________
2. Years of experience in healthcare:

- Less than 5 years
- 5-10 years
- 11-20 years
- 21-30 years
- More than 30 years

1. Type of healthcare setting:

- Secondary care
- Primary care
- Private practice
- Other

**Domain 2:** To assess the level of awareness among healthcare providers in the UK regarding the use of Ayurvedic medicine in the management of chronic diseases.

1. How familiar are you with Ayurveda?

- Very familiar
- Somewhat familiar
- Not very familiar
- Not at all familiar

1. If familiar, which of the following domains of Ayurvedic intervention are you familiar with?

- Herbal medications
- Medications with metals and minerals
- Panchakarma therapies
- Ayurvedic surgical interventions
- Ayurvedic para-surgical interventions
- Dietary interventions
- None of the above

1. Based on comprehensive knowledge of relevant research studies, clinical evidence, and practical experience how much do you understand the efficacy and safety of Ayurvedic medicine in managing chronic diseases?

- Completely understand
- Partially understand
- Have minimal understanding
- Have no understanding

1. In which chronic diseases do you think Ayurveda may be effective in managing?

- Cardiovascular diseases (including heart disease and stroke)
- Chronic respiratory diseases (such as chronic obstructive pulmonary disease - COPD)
- Diabetes (Type 1 and Type 2)
- Musculoskeletal conditions (such as arthritis)
- Neurological diseases (such as Parkinson's disease and multiple sclerosis)
- Gastrointestinal diseases (such as inflammatory bowel disease)
- Kidney diseases (such as chronic kidney disease)
- Liver diseases (such as cirrhosis)
- Mental health disorders (including depression, anxiety, and dementia)
- Cancer
- Other (please specify)
- I’m not sure

1. How much do you agree with the statement 'I'm equipped enough to counsel patients on the benefits and harms of Ayurvedic interventions in chronic disease management ?

- Strongly Agree
- Agree
- Neutral
- Disagree
- Strongly Disagree

1. How easy is it for you to find reliable information regarding Ayurveda?

- Very easy
- Easy
- Neither difficult normal easy
- Difficult
- Very difficult
- I don’t know how to access this information

1. What sources do you typically use to obtain information about Ayurveda?

- Medical journals
- Conferences or seminars
- Online resources
- Colleagues or peers (websites, blogs, forums, etc.)
- Other (please specify)

1. Have you ever undergone training in Ayurvedic medicine or participated in any Ayurvedic medicine courses or workshops?

- Yes, extensively
- Yes, moderately
- Yes, minimally
- No

1. How familiar are you with the legal and regulatory issues related to Ayurveda?

- Very familiar
- Somewhat familiar
- Not very familiar
- Not at all familiar

1. How do you rate the quality of existing scientific research on the efficacy and safety of Ayurvedic medicine in managing chronic diseases?

- High quality
- Moderate quality
- Low quality
- Not sure

**Domain 3:** To assess the attitude of healthcare providers in the UK towards the use of Ayurvedic medicine in the management of chronic diseases.

1. Ayurveda should be included in the treatment options offered to patients with chronic diseases

- Strongly Agree
- Agree
- Neutral
- Disagree
- Strongly Disagree

1. Ayurveda should be accessible via NHS for people living with chronic diseases in the UK?

- Strongly Agree
- Agree
- Neutral
- Disagree
- Strongly Disagree

1. Patients who use Ayurveda for the management of chronic diseases should have access to qualified and trained practitioners.

- Strongly Agree
- Agree
- Neutral
- Disagree
- Strongly Disagree

1. Patients should be informed of both the benefits and risks of using Ayurvedic medicine as a treatment option.

- Strongly Agree
- Agree
- Neutral
- Disagree
- Strongly Disagree

1. The usage of ayurvedic interventions should be regulated by MHRA (Medicines and Healthcare products Regulatory Agency)/ NHS in the UK

- Strongly Agree
- Agree
- Neutral
- Disagree
- Strongly Disagree

1. I’m willing to learn more about the potential role of Ayurveda in chronic disease management

- Strongly Agree
- Agree
- Neutral
- Disagree
- Strongly Disagree

1. I believe that orientation to Ayurveda through educational training should be offered to healthcare professionals.

- Strongly Agree
- Agree
- Neutral
- Disagree
- Strongly Disagree

1. I believe that Ayurveda can be used to prevent the onset of chronic diseases.

- Strongly Agree
- Agree
- Neutral
- Disagree
- Strongly Disagree

1. I believe Ayurveda can help manage chronic health conditions.

- Strongly Agree
- Agree
- Neutral
- Disagree
- Strongly Disagree

1. Ayurveda can be used to address the root cause of chronic diseases rather than just treating the symptoms.

- Strongly Agree
- Agree
- Neutral
- Disagree
- Strongly Disagree

1. I believe that Ayurveda has the potential to improve the quality of life of patients with chronic diseases.

- Strongly Agree
- Agree
- Neutral
- Disagree
- Strongly Disagree

1. I think that Ayurveda has the potential to help patients with chronic diseases who have not been helped by conventional medicine.

- Strongly Agree
- Agree
- Neutral
- Disagree
- Strongly Disagree

1. Incorporation of Ayurvedic therapies into routine clinical practice would result in increased patient satisfaction.

- Strongly Agree
- Agree
- Neutral
- Disagree
- Strongly Disagree

1. I believe that Ayurveda can contribute to patient-centred care.

- Strongly Agree
- Agree
- Neutral
- Disagree
- Strongly Disagree

1. I believe that healthcare professionals should discuss about Ayurvedic medicine with their patients.

- Strongly Agree
- Agree
- Neutral
- Disagree
- Strongly Disagree

1. I would be willing to refer patients for Ayurvedic treatment if it were available and appropriate.

- Strongly Agree
- Agree
- Neutral
- Disagree
- Strongly Disagree

1. There should be a formal referral system in place between conventional healthcare providers and Ayurvedic practitioners.

- Strongly Agree
- Agree
- Neutral
- Disagree
- Strongly Disagree

1. The referral system to an Ayurvedic practitioner should be regulated by the government in UK

- Strongly Agree
- Agree
- Neutral
- Disagree
- Strongly Disagree

1. Healthcare providers should work collaboratively with Ayurvedic practitioners to provide the best care for patients with chronic diseases.

- Strongly Agree
- Agree
- Neutral
- Disagree
- Strongly Disagree

1. I would be willing to collaborate with Ayurvedic practitioners in managing patients with chronic diseases.

- Strongly Agree
- Agree
- Neutral
- Disagree
- Strongly Disagree

1. There should be more research into the efficacy and safety of Ayurvedic medicine for the management of chronic diseases.

- Strongly Agree
- Agree
- Neutral
- Disagree
- Strongly Disagree

1. More resources should be allocated to research the potential benefits of Ayurvedic medicine for the management of chronic diseases.

- Strongly Agree
- Agree
- Neutral
- Disagree
- Strongly Disagree

1. The lack of regulation in the practice of Ayurveda poses a risk to patients with chronic diseases.

- Strongly Agree
- Agree
- Neutral
- Disagree
- Strongly Disagree

1. Healthcare providers should work with Ayurvedic practitioners to develop evidence-based guidelines for the management of chronic diseases.

- Strongly Agree
- Agree
- Neutral
- Disagree
- Strongly Disagree

1. Ayurvedic interventions should be funded via the National Health Service (NHS) in the UK

- Strongly Agree
- Agree
- Neutral
- Disagree
- Strongly Disagree

1. In your opinion, what should be done to ensure the safe and effective use of Ayurveda in the UK, assuming a consensus among the public?

- Develop guidelines and regulations
- Educate healthcare providers and patients
- Allocate more resources in facilitating the use of Ayurveda
- Collaborate with Ayurvedic practitioners and organizations to promote greater transparency and accountability in the industry
- Conduct more research on Ayurvedic interventions
- Other (please specify)

**Domain 4: To assess the current practice of healthcare providers in the UK in incorporating Ayurvedic medicine in the management of chronic diseases.**

1. How often do you perceive interest from patients in discussing Ayurvedic interventions with you?

- Frequently
- Occasionally
- Rarely
- Never

1. If a patient expresses interest in using Ayurveda, how do you typically respond

- Provide information
- Refer the patient to an Ayurvedic practitioner
- Offer to work with an Ayurvedic practitioner to provide care for the patient
- Discouraged them from seeking Ayurvedic treatment
- Don’t offer any definitive advice
- Other (please specify)

1. In your professional capacity, how comfortable are you in communicating with your patients regarding Ayurvedic interventions?

- Extremely comfortable
- Moderately comfortable
- Slightly comfortable
- Not at all comfortable

1. With approximately what percentage of your patients do you talk about possible benefits of using a Ayurvedic treatment?

- 0%
- 1–25%
- 26–50%
- 51–75%
- 76–100%

1. With approximately what percentage of your patients do you talk about possible harmful outcomes of using Ayurvedic interventions?

- 0%
- 1–25%
- 26–50%
- 51–75%
- 76–100%

1. Who usually initiates discussions of benefits and risks of a Ayurvedic intervention?

- I initiate the discussion
- Patient initiates the discussion
- Sometimes I and sometimes the patient initiates the discussion
- Third party initiates the discussion
- Not Applicable

1. Do you routinely ask patients whether or not they are using Ayurvedic interventions as part of their healthcare?

- Yes, always
- Yes, sometimes
- No, rarely
- No, never
- Not applicable to my practice

1. How often do you think patients report using Ayurveda without disclosing it to you?

- Frequently
- Occasionally
- Rarely
- Never

1. How likely is it that you would refer a patient to an Ayurvedic practitioner for treatment of a chronic disease?

- Extremely likely
- Somewhat likely
- Neither Likely Nor Unlikely
- Somewhat Unlikely
- Extremely Unlikely

1. Have you ever referred a patient to an Ayurvedic practitioner?

- Yes
- No

1. If yes, was your reason for referral?

- The patient expressed an interest in Ayurvedic interventions
- Ayurvedic interventions were recommended as a complement to allopathic treatments
- The patient had a chronic condition that was not responding well to allopathic treatments
- Other (please specify) _______________

1. If no, what has stopped you from referring patients to an Ayurvedic practitioner?

- Lack of knowledge
- Concerns about safety
- Lack of evidence for efficacy
- Cost
- Lack of access to Ayurvedic practitioners
- Other (please specify)

1. If you had considered referral, how easy is it to access Ayurvedic practitioners for patient referrals?

- Very easy
- Somewhat easy
- Not very easy
- Not at all easy
- Not applicable

1. I have observed positive outcomes with Ayurvedic treatment. (personal experience or on the experiences of patients)

- Yes
- No

1. What do you perceive as the biggest challenge to using Ayurveda for chronic diseases in the UK?

- Lack of scientific evidence
- Lack of regulatory framework
- Limited understanding of Ayurveda among conventional healthcare providers
- Limited patient demand
- Limited resources
- Other (please specify) ____________

1. Do you think the current regulatory framework is sufficient to ensure patient safety?

- Yes
- No

1. What changes, if any, would you like to see in the regulatory framework for Ayurvedic practice in the UK?
2. Do you think there are enough resources available to support the use of Ayurveda into healthcare in the UK?

- Yes
- No

1. If no, what types of resources are lacking?
2. What steps do you think can be taken to overcome the challenges to using Ayurveda in the UK ? (Select all that apply)

- Increase scientific research on Ayurveda
- Develop a regulatory framework for Ayurveda
- Provide education and training to conventional healthcare providers
- Increase awareness among patients
- Allocate more resources
- Other (please specify) ____________

1. How often do you discuss Ayurveda with other healthcare providers (e.g. colleagues, specialists)?

- Frequently
- Occasionally
- Rarely
- Never

1. How often do you perceive conflict between Ayurveda and conventional medicine paradigms?

- Frequently
- Occasionally
- Rarely
- Never

1. How often do you perceive lack of acceptance from colleagues regarding use of Ayurveda for chronic disease management?

- Frequently
- Occasionally
- Rarely
- Never

1. Would you be willing to collaborate with Ayurvedic practitioners in patient care?
   - Yes
   - No
